# Lessons from the contrast dye shortage during COVID-19: A narrative review

**DOI:** 10.1097/MD.0000000000032286

**Published:** 2022-12-23

**Authors:** Christiaan Yu

**Affiliations:** a Department of Respiratory Medicine, Alfred Health, Melbourne, Australia; b Central Clinical School, Monash University, Melbourne Australia.

**Keywords:** contrast, COVID-19, CT, healthcare systems, radiology department

## Abstract

The sudden contrast dye shortage, precipitated by a temporary forced closure of healthcare plant, has limited the supply of iodinated contrast media to Australia. Furthering the impact of the coronavirus disease 2019 pandemic, this new crisis has increased burden on the radiology system. Lessons from the strategies applied during the shortage should be used as building blocks as safeguards for the future. A pragmatic approach to education and training is required in an ever-changing environment. Our relationships between medical specialties and manufacturers are paramount to maintaining an effective workflow. An ongoing commitment to a strong workforce will be the backbone to overcome another challenge in these uncertain times.

## 1. Introduction

### 1.1. COVID 19 and the impact on radiology services

The unprecedented coronavirus disease 2019 (COVID-19) pandemic has severely disrupted all facets of life. From healthcare to agriculture, there has not been a sector that has not experienced the ill effects of the prolonged pandemic.^[1]^ In addition to morbidity and mortality associated with this highly transmissible virus, a significant proportion of the negative consequences were a result from a range of policies introduced to slow the transmission of COVID-19. The early restrictions were based on the tenets of protection via masks, hygiene, physical distancing and isolating in the presence of any respiratory or systemic symptoms. As the initial endemic progressed to a global pandemic, further limitations were put into place. Non-essential businesses and industries had to temporarily shut down and members of the public were encouraged to stay home. Furthermore, travel restrictions tightened the visitors in and locals out of Australia. Though these strict measures were deemed necessary from a public health standpoint, there were negative consequences in other areas of the economy. There was an unpredictable and irregular cycle of lockdown and reopening as COVID-19 cases began to fluctuate and new subvariants such as Omicron and Delta emerged. While these mutations did not confer a more severe disease, the transmissibility was higher than the original coronavirus. The contagious subvariants led to a high proportion of healthcare workers off sick and some experiencing long term illness impacting their ability to return to work.^[2]^ COVID-19 is now recognized as an occupational disease with implications for healthcare systems and workflow.

Radiology services were one of the areas severely impacted by the pandemic. Large scale restructuring was required to meet the needs of COVID-19. Even though non-urgent presentations and imaging were delayed, there was a large upscale in patients with respiratory tract or systemic systems presenting for the radiological diagnosis or severity of COVID 19.^[3]^ Thrombotic events, predominately pulmonary emboli, occurred in up to one-third of patients with COVID-19 and is associated with increased morality.^[4]^ The added requests for computed tomography (CT) pulmonary angiogram and lung ventilation perfusion scan strained an already stretched service. The department also had to account for the provision of service to COVID-19, non-COVID-19 and also COVID-19 at-risk patients. This new classification led to widescale change and the need for standardized protocols to ensure technicians, cleaning staff and clinicians were able to clean and disinfect equipment.^[5]^ Contact surfaces required a greater frequency of cleaning with sodium hypochlorite or phenolic liquids. If a patient initially did not have any suspicious symptoms but subsequently became suspected or confirmed of COVID-19, a deep clean was required. A thorough decontamination of high-touch surfaces and objects increased workload and results in delays to service. Moreover, air purifiers and ultraviolet lamps were introduced to ensure the environment remained safe. Radiology departments, in collaboration with hospital infection control and the chief health officer had to collaborate in dynamic circumstances and guidelines reviewed on a regular basis. Overall, the increased demand and unrecognizable workflow had a profound impact on radiography services around the world.^[6]^

## 2. Contrast media shortage

### 2.1. Unexpected contrast shortage and management

In June 2022, because of ongoing COVID-19 lockdown, a shutdown in a large healthcare manufacturing facility in one of the major suppliers led to a global shortage of iodinated contrast media (ICM). While this only lasted for a few weeks, this acute and severe shortage affected millions of examinations and resulted in an unexpected imaging crisis in radiology departments. Alternate ICM agents such as a different form of iodixanol, iopamidol, iopromide and ioversol were rapidly depleted as institutions began searching for alternative agents and stockpiling. All modern intravenous contrast agents are now iodine based. ICM are used in imaging techniques to enhance anatomical differences between soft tissue densities on various forms of imaging particularly CT. The iodine causes an increased absorption and scattering of the delivered radiation which in turn increases the attenuation of an organ or tissue. Contrast is generally safe and well tolerated although a small proportion of patients may experience adverse effects and a smaller subset may not be suitable for this scan. The use of contrast in CT has doubled in the past few decades and used in almost half of all CT scans performed.^[7]^ Hence, several ICM conversation strategies were implemented to work through the supply shortage.^[8]^

The first solution was to delay the scan and this revolved around the urgency and necessity of the exam. Deferment of low risk scans such as follow-up of incidental findings or annual surveillance without a change in clinical status were utilized in the most critical of shortages. As this step was not completely risk free, manual review of initial imaging request and discussion with referring clinician was frequently undertaken. If it was necessary to perform imaging in prompt fashion, the next step was to determine if it could be changed to a non-contrast CT scan. Apart from scans for suspected stroke, dissection, level 1 trauma (most serious injuries that required large trauma teams and fast response times), cardiac catheterization and select malignancies, examinations may be performed without contrast to answer the clinical question. Assessing most pulmonary parenchymal, interstitial, airway, pleura and infective respiratory conditions do not require the use of contrast. Focus is placed rather on the thickness of scans, positioning (supine or prone) and phase of inspiration.^[9]^ Similarly, alternate modalities of imaging could be selected if it could confirm the diagnosis pursued. Nuclear medicine scans can be used for the diagnosis or workup of pulmonary embolism, pyrexia of unknown origin, staging of select cancers and gastrointestinal bleeding. Contrast enhanced ultrasound can also be used in the evaluation for focal hepatic or cystic pancreatic pathologies.^[10]^ First generation ultrasound contrast agents comprised of microbubbles of air that were dissolved in blood when subjected to pressures in the sonography field. Subsequent contrast agents contained microbubbles of perflurocarbon, sulfur hexafluoride stabilized in phospholipid membranes or nitrogen gas.

If it is established that a contrast CT was required to be performed, the amount of ICM could be reduced through weight-based dosing and lowering the tube voltage. A level of diagnostic image quality can be retained and amount of radiation can also be reduced through these measures.^[11,12]^ However, in the extremes of body mass index and extent of pathology these may not be feasible. Scanner software and hardware are also important considerations to prevent untenable images. Depending on the indication, hospital default ICM could be swapped to a traditional ionic agent. Carbon dioxide angiography which is commonly used from those with chronic kidney disease or previous immunoglobulin E-based allergic reaction can be used for peripheral arteriography.^[13]^ Finally, correlating dose protocols to vial volumes available prevents excessive ICM waste.

## 3. Reshaping the future of radiology services

### 3.1. The referrers

This global ICM crisis precipitated by COVID-19 has presented us with an invaluable opportunity to reshape the future of radiology service at all levels. From a grassroots level, we need to start by targeting the source and foundation of radiology requests – the referrers. Close collaborations should be formed with primary care, critical care units including emergency and intensive care units, ambulatory care and hospital wards. Hospital specific policies or protocols should be established to clearly delineate the necessity of ICM for a scan based on a particular or variety of diagnosis in the context of the clinical case. Particular emphasis and education can be tailored to specialties that commonly require forms of contrast which are cardiology, respiratory, vascular surgery, general surgical, gastroenterology, urology and oncology in both its medical and radiation forms. The complexities of the current pandemic have made a detailed and clear history in the original request a necessity. Constant lines of communication through a direct channel or via a specialty-based radiology meeting should occur when ordering complex examinations. The role of multidisciplinary team (MDT) meetings has been highlighted to provide a standard methodology when approaching complex pulmonary disorders.^[14]^ In addition, MDT facilitates the discussion of complicated patients and allows each invested unit to input their recommendation, providing a targeting patient therapy.^[15]^ Radiology should have an invested role in these MDT to improve the efficiency and coordination when making imaging modality selections for our patients. The ever-evolving pandemic has added not only both the volume, but also the complexity of referrals. The use of the MDT, automatically, seems to be a highly useful and powerful tool to identify the need for ICM at the front end. The short term ICM shortage has presented various medical specialties the unique occasion to experience a radiology role in parallel to their own.

### 3.2. The trainees

Before attaining specialist qualifications and being recognized as a radiologist, trainees must undergo a highly structured training program with core work-based assessment over a period of 4 to 6 years. The COVID-19 pandemic has severely disrupted the typically streamlined process of radiology training. Re-deployment from usual rotations and suspension of non-essential procedures have limited radiology trainee’s skill acquisition and potentially interrupt progression of training. As workload demand increases, rates of reporting discrepancies rise as well.^[16]^ Factors identified included high proportion of reporting time per shift and junior level of training. In hospitals, after-hours services are generally run by radiology trainees. Preliminary reports are generated, and further vetting occurs in the following workday by a senior radiologist. With the current ICM crisis and limitations, it will be imperative to evaluate the extent and nature of discrepancies in these reports.

At the height of lockdown, pilots honed their aviation skills through simulators. Race car drivers have seen certain competitions replaced by a virtual format. In clinical medicine, simulation training has been present for a few decades and assists with procedural skills and situational awareness.^[17]^ Trainees should be able to utilize such programs to report in a safe virtual environment with the ability to toggle through amount of ICM used. These skills can be assessed pre-specified benchmarks and may offer a validated form of clinical experience. Training models that can bypass some of the limitations placed by COVID while providing risk-free scenarios such the ICM one. Radiology-based training programs that can mimic real life unexpected crisis in the form of a contrast shortage, lighting malfunction or difficult patient could be vital to future training programs.

### 3.3. The backlog

Following this shortage, there will be delayed or canceled tests that would require rescheduling in due course. This backlog of referral threaten to jeopardize all health outcomes. COVID-19 has already seen the increase in X-ray and CT compared to all other modalities worldwide.^[18]^ Compounded by further factors of reduced productivity from staff burnout and fatigue from year-long pandemic, the ICM shortage will generate a larger burden to shoulder. Hospital services and healthcare systems have to identify factors to reduce the backlog and use strategies to overcome this.

Workforce and staffing are the vanguard of any solutions. Rediverting professionals based on current and predicted service needs will be a step to restoring previous standard of care. Optimizing work conditions and staff retention programs via financial or other means should be considered. In hospitals based in Melbourne Australia, staff have been offered a payment of $3000 AUD to all staff employed in public health services and ambulances for those employed in the winter months of 2022. Wellbeing programs have expanded to provide free meals and refreshments to staff working late evening and night shifts. By tailoring to the individual health provider’s needs, these services should be given flexibility to deliver such programs.

The importance of the radiology service as a backbone of any healthcare department has not been clearer. Budgets and projections for short, medium and long term need to invest in infrastructure and new models of care. Monitoring current policies to rationalize radiology utilization and reduction of ICM waste to decrease inequalities will have to continue. The division of patients based on their COVID-19 status and risk results in the separation of care. The possibility of outsourcing non-urgent tests to non-tertiary centers that have availabilities ought to be considered. Prioritizing and ensuring patients are booked to fit supply capacity without overfilling the waiting rooms will be a fine balance.

### 3.4. The suppliers

We should also be cognizant of the factory closure which precipitated the lockdown and impact on local workers and economy. The symbiotic relationship between ICM and healthcare service should be strengthened during this time. Although the production factories may be located far away, working with resource groups can allow institutions to take part in programs outside their regular scope and provide forms of support. Healthcare networks can develop ongoing partnerships with medical supply manufacturers as they have a shared common goal of providing timely, quality care and service to the community. Strong lines of communication through regular meetings or correspondence may allow the foreshadowing of any potential issues to arise from the either end. Even though some events are unpredictable, understanding the landscape of local events can be helpful predicting possible shortcomings.

Moreover, the hospital/clients’ input can promote change. An example would be radiology departments working with manufacturers to provide insight into the optimal vial volume. Currently, single-use vials should not be used for >1 examination due to infection risks. An optimal production of vial sizes would take into account hospital protocols as well as production capability to determine the very best use of resources.

### 3.5. Future directions

Since radiology was first noted as an entity in 1920, the specialty has advanced markedly through innovation of technology. Facing an ICM crisis in the middle of a pandemic truly is a true test of the resilience and resolve of front-line workers and the radiology service. The current challenge is to establish a safe way of working through the reduced supply of contrast. There is an opportunity to demonstrate strong leadership skills by creating and adapting protocols and guidelines to ensure maximum efficiency and protecting our patients.

Moving forward, radiology services, through working with healthcare systems, need a clearly defined and aligned strategy to enable future planning. Reported success or failures will have to be measured following this crisis to identify areas for improvement by addressing shortcomings and potential weaknesses. Furthermore, this is a chance to mold the way radiology as a specialty interacts with other services as well as how they interact with us.

It is almost 3 years into a pandemic and workload remains at an all-time high as many departments and services have returned to pre-pandemic times. The rippling effects on the global, social and individual scale of COVID-19 will likely continue for years to come. Safeguards have to be put in place in time for the inevitable next variant, endemic or pandemic. They must incorporate disaster plans and be able to be activated quickly and address staffing issues. However, this is by no means an easy task. Managing backlogs may derail short term plans and there needs to be a balance of high acuity cases and ones already delayed.

Finally, the ICM shortage may have exposed some of our frailties as a service but this can be looked upon as opportunities for quality improvement. Our mission to provide patient-centered care as well as optimizing professional development, engagement and wellbeing of our staff must be at the forefront of any improvement strategy. Through this unexpected journey, we can all further develop a more advanced radiology service for the future.

## Author contributions

**Conceptualization:** Christiaan Yu.

**Writing – original draft:** Christiaan Yu.

**Writing – review & editing:** Christiaan Yu.
